# Household Food Waste Quantification and Cross-Examining the Official Figures: A Study on Household Wheat Bread Waste in Shiraz, Iran

**DOI:** 10.3390/foods11091188

**Published:** 2022-04-19

**Authors:** Shahin Ghaziani, Delaram Ghodsi, Karsten Schweikert, Gholamreza Dehbozorgi, Shiva Faghih, Shabnam Mohabati, Reiner Doluschitz

**Affiliations:** 1 Computer Applications and Business Management in Agriculture, University of Hohenheim (410C), 70593 Stuttgart, Germany; reiner.doluschitz@uni-hohenheim.de; 2 National Nutrition and Food Technology Research Institute, Tehran 19816-19573, Iran; delaramghodsi@yahoo.com; 3 Department of Nutrition Research, Faculty of Nutrition Sciences and Food Technology, Shahid Beheshti University of Medical Sciences, Tehran 19816-19573, Iran; 4 Core Facility Hohenheim (CFH), University of Hohenheim (640), 70593 Stuttgart, Germany; karsten.schweikert@uni-hohenheim.de; 5 Horizon Smart SAT (Surveillance and Analysis Technology), Fars Science and Technology Park, Shiraz 71976-87811, Iran; r.dehbozorgi.1988@gmail.com; 6 Department of Community Nutrition, School of Nutrition and Food Sciences, Shiraz University of Medical Sciences, Shiraz 71348-14336, Iran; shivafaghih@gmail.com (S.F.); mohabati_sh@yahoo.com (S.M.); 7 Nutrition Research Center, Shiraz University of Medical Sciences, Shiraz 71348-14336, Iran

**Keywords:** food loss and waste, food waste accounting and data, food waste index, sustainability, sustainable production and consumption, food waste quantification, global data

## Abstract

The global consumer food waste (FW) estimates are mainly based on modeling data obtained from governments. However, a major data gap exists in FW at the household level, especially in developing countries. Meanwhile, the reliability of the existing data is questionable. This study aimed to quantify wheat bread waste (HBW) in Shiraz, Iran, and cross-examine the governmental HBW data. Face-to-face waste recall questionnaire interviews were conducted in 419 households from December 2018 to August 2019. A multistage sampling strategy consisting of stratification, clustering, and systematic sampling was employed. Moreover, we carried out a comprehensive document review to extract and analyze the official HBW data. The results revealed that the HBW in Shiraz is 1.80%—the waste amounts for traditional bread and non-traditional bread were 1.70% and 2.50%, respectively. The survey results were compared with the previous official data, revealing a substantial contradiction with the 30% HBW reported between 1991 and 2015. Possible reasons for this disparity are explored in this paper. Although our results cannot be generalized to other food commodities and locations, our findings suggest that considering the substantial likelihood of bias in the official data, policymakers should conduct more FW measurements and re-evaluate the accuracy of the existing data.

## 1. Introduction

Despite the tremendous planning and efforts to ensure global food and nutrition security, the food production and consumption system appears to be inefficient and unsustainable. The members of the United Nations (UN) have agreed to end hunger while preserving the environment by 2030 through sustainable development goals (SDG) number 2 (zero hunger) and 12 (responsible consumption and production) [1]. Based on this rationale, avoiding or reducing food loss and waste (FLW) is prudent to increase food security while reducing the environmental and economic burdens. The third target of SDG 12 is explicitly focused on the reduction of FLW [1].

In order to plan the most effective courses of action toward achieving the UN’s FLW reduction target, gaining a realistic and accurate understanding of the status quo in the agri-food system plays a vital role. Furthermore, accessing robust and accurate data is the key to monitoring and assessing whether an FLW reduction plan achieves its objectives [2]. Two UN-affiliated organizations—i.e., the Food and Agriculture Organization (FAO) and the United Nations Environment Programme (UNEP)—are responsible for monitoring SDG 12.3 and providing information and guidance for the decision makers to develop FLW reduction policies [2].

It is important to distinguish food waste from food loss, as their stages of occurrence and quantification methods are fundamentally different. According to the FAO’s definition, food loss refers to the reduction in the amount of food along the production and supply chains from farm to, but not including, retail, whereas food waste is the discarding of food at the consumption stage [3]. The consumption stage includes retail, foodservice, and households [4]. The terms “food loss” and “food waste” are hereafter used according to the definitions mentioned. Currently, the FAO is focused on food loss, and the UNEP is the custodian for food waste [2].

In 2011, when the FAO was solely responsible for both food loss and food waste, the first systematic analysis of global FLW showed that about one third of the total food produced is lost or wasted throughout the food supply chain (FSC) [5]. The FAO’s 2019 report on FLW indicated the food loss to be 14% [2], and, in 2021, the UNEP estimated that 17% of globally produced food is wasted [4]. These figures add up to 31% of FLW, which strongly agrees with the FAO’s 2011 report. This means that the FLW reduction plans have not been proceeding in the UN’s desired direction since 2011.

However, according to the FAO’s 2011 report [2], the lack of data on the extent and locations of FLW occurrence is substantial. Between 1990 and 2017, the FAO received FLW data annually from only 39 countries [2]. Therefore, the question arises as to what extent the recent estimations made by the FAO and UNEP reflect the actual amount of FLW, as their findings rely heavily on estimations and exploitation of the limited data available. Therefore, it is crucial to clarify how these organizations currently gather FLW data.

The FAO has extensively conducted studies on food loss in different countries during the last few years. Examples may include but are not limited to the analysis of cassava loss in the Republic of Guyana [6], studies on maize and rice losses in the Democratic Republic of Congo [7], case studies on postharvest loss of chickpea, mango, and rice in India [8], and groundnut and maize loss analyses in Malawi [9]. However, the origins of most of the FAO’s food loss statistics are still governments and official local sources [3]. At the same time, the UNEP receives food waste data almost entirely from local governments [4] and, to the best of our knowledge, has no ongoing study on food waste. Therefore, the food waste estimates mainly rely on proxy data, resulting in a more substantial data gap in food waste compared to food loss [4,10].

Presumably, quantifying food waste seems more challenging compared to food loss. The argument would seem to be that analyzing the mass flow along FSCs to evaluate food loss may seem relatively straightforward, although it entails transparency. Notably, countries with advanced infrastructure keep precise records of food products throughout their FSCs—e.g., the European Union (EU) countries [11]—and can better estimate food loss. On the other hand, evaluating food waste may seem more challenging because monitoring the material flow at the consumption stages is complicated, especially household food waste (HHFW), which accounts for 61% of the total food waste at the consumption stage [4]. Generally, in most countries, a form of food inventory management is expected to exist in the retail and foodservice sectors, which may facilitate measuring food waste using various methods, such as mass balance, volumetric assessment, and counting/scanning [4]. However, the multifaceted nature of the HHFW complicates its quantification.

The UNEP implements a three-level methodology—i.e., level 1: modeling the available data; and levels 2 and 3: food waste measurement and providing additional information [4]. Currently, the UNEP estimates are mainly based on modeling data, and measurements are the future plans. The UNEP analyzed the food waste data from 59 countries, only 17 of which used standard food waste quantification methods, yielding “high-quality data compatible with SDG 12.3” [4] (p. 9). In the case of the other 42 countries, where there is a lack of robust data, the UNEP approximately estimated food waste by using the modeling approach to extrapolate the data provided by other countries [4]. In particular, a substantial data gap exists at the household level [4]. Given that the UNEP’s success in providing proper guidance toward SDG 12.3 depends on achieving a precise estimation of HHFW, the importance of evaluating the accuracy of the data provided by local governments and identifying the main data gaps cannot be emphasized enough.

During the last decade, many studies focused on HHFW quantification. However, according to Xue et al.’s [10] systematic review, the majority of these studies were carried out in developed countries, leaving a data gap in developing countries. A similar conclusion can be drawn from the UNEP’s 2021 report [4]. According to the UNEP, only 14 countries—all developed countries except Ghana—have high confidence data on HHFW. Distinctively, the Near East and North Africa (NENA) region countries lack reliable data on HHFW [4,12,13].

The NENA region imports over half of its food and still struggles to meet the demand while its level of FLW is estimated to be above the global average—annually 250 kg per person [14]. In many NENA countries, food is mainly lost at the production and postharvest stages, basically due to poor technological infrastructure [14]. However, a substantial share (32%) of the FLW still occurs at the consumption stage [15]. The rich NENA countries, such as the Kingdom of Saudi Arabia (KSA), waste even more food at the consumption stages, mainly due to their extravagant lifestyles and cheap food [16]. Based on the 2021 report of the Food Sustainability Index [17], the food waste amounts in KSA and the United Arab Emirates (UAE) are estimated to be 151 and 134 kg per person per annum, respectively.

However, despite the high level of food waste, after Sub-Saharan Africa, NENA has the lowest policy response to food waste [17]. A major factor hindering the food waste reduction plans in most NENA countries is known to be the lack of reliable data [14]. A systematic review from 2020 [18] accentuated the scarcity of FW data in some of the Arab states of the Persian Gulf. The UNEP has classified the confidence level of the different countries’ HHFW estimates into “high confidence”, “medium confidence”, “low confidence”, and “very low confidence” [4] (p. 12). Among the NENA countries, only KSA has “high confidence” data, and Bahrain, Iraq, and Lebanon have “medium confidence” data [4] (p. 65). The HHFW data of Jordan, Kuwait, Mauritania, Oman, Palestine, Qatar, Syria, and UAE are classified as “low confidence” [4] (p. 65). All other north African countries, along with Yemen and Iran, have “very low confidence” HHFW estimates [4] (p. 65).

As a major country in NENA, Iran faces tremendous challenges in quantifying and reducing its food waste. Over the last decade, Iran’s economy has been shrinking drastically due to international sanctions. Between 2011 and 2015, the country’s crude oil exports almost halved [19]. The U.S. “maximum pressure” policy cut Iran’s access to international financial services [19]. Hence, Iran has been overusing its natural resources to overcome international sanctions and reach self-efficiency [20,21]. On the other hand, Iran’s young and dynamic population seems to be transitioning toward a modern and extravagant lifestyle with a tendency toward consumerism, resulting in increasing food waste [5]. Based on the UNEP data, HHFW in Iran is around 5.9 million tons per annum but, as mentioned earlier, with very low estimation confidence [4]. Studies on HHFW in Iran were mainly focused on food waste attributions rather than its amount [22,23,24]. Therefore, information on the quantity of HHFW in Iran is scarce.

The present study aimed to quantify HHFW in Iran and critically examine the accuracy of governments’ HHFW statistics by comparing them with our primary data. Due to limited research capacities, investigating all food commodities was not possible. Therefore, wheat bread (hereafter referred to as bread) was chosen as the focus of the study due to its importance as the primary staple and most consumed food commodity in many developing countries, especially in the NENA region [25].

NENA countries are among the biggest wheat importers globally, with around 36 million tons of wheat per annum [14]. Bread is considered to be of utmost importance to Iran’s economy and is the most popular food commodity in the country [26,27]. Iran is among the biggest wheat producers worldwide. The FAO’s latest official data demonstrate that Iran produced more than 13 million tons of wheat in 2018, ranking 15th globally [28]. No recent study can be found reporting the Iranians’ bread consumption amount. However, the last and most commonly cited national investigation, which was based on the data from 2000 to 2002, indicated that the average daily bread intake in Iran was 320 gr per capita [29]. Jafari et al. [30] assumed that Iran’s bread consumption should be similar to that of Turkey—supposedly due to cultural and geographical similarities—and, therefore, one of the highest in the world. A 2012 study on 22 European countries, the United Kingdom, and Turkey revealed that Turkey’s average per capita bread consumption is 411 gr per day—more than 2.5 times higher than the average of the European countries [31].

To achieve the aim of this study, a survey was conducted to quantify household wheat bread waste (HBW) in Iran. The study location was chosen to be Shiraz—home to about 1.6 million inhabitants [32] and the capital of Iran’s major wheat-producing province, Fars. Among 31 provinces, Fars produces almost 10% of the country’s wheat—about 1.2 million tons per year [33]. Because official statistics on HBW were not easily accessible, we carried out a thorough document review to gather, analyze, and summarize the previous reports on HBW in Iran.

## 2. Materials and Methods

### 2.1. Study Design and Sampling

The present study was carried out from December 2018 to August 2019 in Shiraz, Iran. A door-to-door self-assessment recall questionnaire survey was conducted to quantify the HBW and gather other relevant data. A total of 419 households were surveyed. In this study, a ‘household’ was defined as two or more persons living in one house and sharing food and the costs for food. One person per household, identified as the member responsible for food preparation and nutrition, was interviewed. The study was performed in line with the Declaration of Helsinki, and the study protocol was approved by the Ethical Committee of the Shiraz University of Medical Sciences, with the code IR.SUMS.REC.1397.595. All interviewees were assured about the anonymity and confidentiality of their responses and provided written consent for inclusion prior to the onset.

The sample size was computed as described below based on Daniel’s equation [34], which is commonly used in population studies. We chose this equation to obtain adequate observations for statistical inference while controlling the survey’s executive costs. Finally, a 10% buffer was added to the computed sample size to account for possible data loss.

$$n=\frac{NZ^{2}P\left(1-P\right)}{\left(N-1\right)E^{2}+Z^{2}P\left(1-P\right)}=384$$

where n identifies the sample size;

*N* is the population (households in Shiraz = 477,916 [32]);

*Z* denotes the *Z* score based on the level of confidence (for a level of confidence of 95%, *Z* = 1.96);

*P* stands for the expected prevalence or proportion (assumed to be 50%);

*E* is an abbreviation for the margin of error (assumed to be 5%).

Final sample size = n + ~10% = 419

A three-layer sampling strategy was implemented to ensure homogeneity in the geographical distribution of the selected samples. In the first instance, stratified sampling was used. Each of the ten municipal districts of Shiraz was defined as one stratum. The number of samples within each stratum was determined using the population weight of each district based on the number of households living there. The latest available national census data were used for reference [32]. In the second instance, cluster sampling was applied. Each district (stratum) was divided into congruent square blocks using the fishnet tool in ArcMap 10.4.1 [35]. A shapefile population map—provided by the city hall of Shiraz—was used to identify the residential blocks. The clusters were randomly selected from the residential blocks within each stratum. The number of clusters was calculated as 10% of each stratum’s sample size. Finally, the target households were selected systematically. Every third house was selected, starting with the house located at the southwest endpoint of the map and spirally approaching the other houses clockwise toward the center of the block until ten households within each cluster were successfully interviewed. If a household was unavailable, the fifth household forward, then backward, was approached. If none of them were available, they were skipped, and the subsequent third household was approached.

### 2.2. Questionnaire Structure and HBW Measurement

A researcher-made questionnaire was used to gather data. The questionnaire had three sections: demographics and socioeconomic data, bread waste, and bread storage condition and duration. The first section consisted of questions on the interviewee’s age, gender, and education level and the occupation of the head of the household. In the second section, questions on the amount of bread purchased and wasted were asked for different types of bread. The last section involved questions about the condition and duration of bread storage in households.

The focus of this study was on ten commonly consumed wheat bread types that were chosen based on the Iranian National Standardization Organization (INSO) [36,37]. The bread types were categorized into traditional bread (TB) and non-traditional bread (NTB). Table 1 presents the bread types’ names and descriptions. Hamburger bread is hereafter referred to as bun. Detailed characteristics and specifications of these bread types are provided in INSO [36,37] and Karizaki [26].

The interviewees were asked to provide an estimation of the number of bread pieces bought in a typical grocery purchase and an estimation of the amount of waste from the same specific purchase. Corrado and Ardente’s [38] (p. 849) definition of “avoidable food waste” was referenced to identify HBW, which refers to the disposed of edible parts of bread not used for other beneficial purposes. These questions were separately repeated for each bread type. In the questionnaire, the HBW amount was stated using the guideline for standard domestic food portion sizes—i.e., one hand palm for a flatbread; a 7 cm cut for a baguette, sandwich, or broetchen; a half of a piece for a bun; and a slice for toast bread [39]. The same guideline was used to convert the wasted portions to grams. A nutritionist calculated the mass using dietary assessment exchange lists if the interviewee’s answer was not expressed using the standard portion sizes. Responses that were too subjective were excluded from the dataset. The mass amount of purchased bread pieces was calculated in grams according to Ghafarpour’s [39] guideline. The waste amount was calculated as the ratio of HBW mass to purchased bread mass, expressed as a percentage. The total HBW was calculated as the mean of all bread types’ waste, as TB and NTB waste were the means of the traditional and non-traditional bread types, respectively.

Questions regarding storage methods and duration were asked regardless of the bread type. The interviewees were asked a multiple-choice question about whether they store bread in a freezer, a refrigerator, or at room temperature. The answers to the storage duration question were grouped into ‘up to 2 days’, ‘3–4 days’, ‘5–7 days’, and ‘more than a week’.

### 2.3. Statistical Analysis

The statistical analyses were performed using IBM SPSS Statistics version 25 [40] and R version 3.6.2. [41] with a significance level of *p* < 0.05. Paired samples and independent samples T-tests were performed to identify significant differences across the bread categories. The linear regression model and ANOVA were used to determine whether the storage method, storage duration, or their interaction significantly affected the HBW amount. The amounts of HBW in the different storage methods and duration groups were compared using a pairwise Bonferroni post hoc test.

### 2.4. Document Review

A comprehensive internet search was carried out to find publications and reports focused on HBW in Iran. The keywords ‘household bread waste’ in Persian and English were used to search within governmental, organizational, and academic reports as well as peer-reviewed articles and conference proceeding papers, without publication date limitation. The transcripts were analyzed to gather information on their bread waste results and methodological approaches.

## 3. Results

### 3.1. Overview of the Surveyed Households

A total of 1548 individuals lived in the 419 surveyed households, with an average household size of 3.69 (SD = 1.22). Table 2 provides a summary of the demographic and socioeconomic descriptions of the surveyed households. It is apparent that the majority of respondents were female, and it is also indicated that females were predominantly responsible for food preparation in the households. On the other hand, most of the studied households were male-headed. Moreover, Table 2 provides information on the education level and occupation of the heads of the households.

### 3.2. Bread Waste

Out of 419 surveyed households, three respondents did not answer the bread wasting and purchasing questions (0.72% missing). Table 3 shows the mean HBW percentage presented for the different bread types and categories. The NTB waste numerical value was almost 1.5 times higher than TB waste. A paired sample T-test did not find any significant difference between the two categories. However, as not all households were NTB consumers, the paired comparison excluded around 27% of the TB waste observations. Therefore, an independent samples T-test was carried out, which revealed that the difference between TB and NTB waste was significant (α = 0.016). Lavash and baguette had the highest waste in their bread categories.

The number of observations in Table 3 also indicates how many households consumed each bread type. The bread with the highest consumption was Sangak, while Taftoon was the least consumed. A total of 210 of the interviewees (50.48%) claimed that they do not waste bread at all in their households. Out of 416 TB-consuming households, 237 (56.97%) reported being zero-wasters, while 201 out of 304 NTB-consuming households (66.12%) were zero-wasters. The mean percentages are presented, taking the zero-waste values into account.

### 3.3. Storage Method and Duration

**Figure 1:**
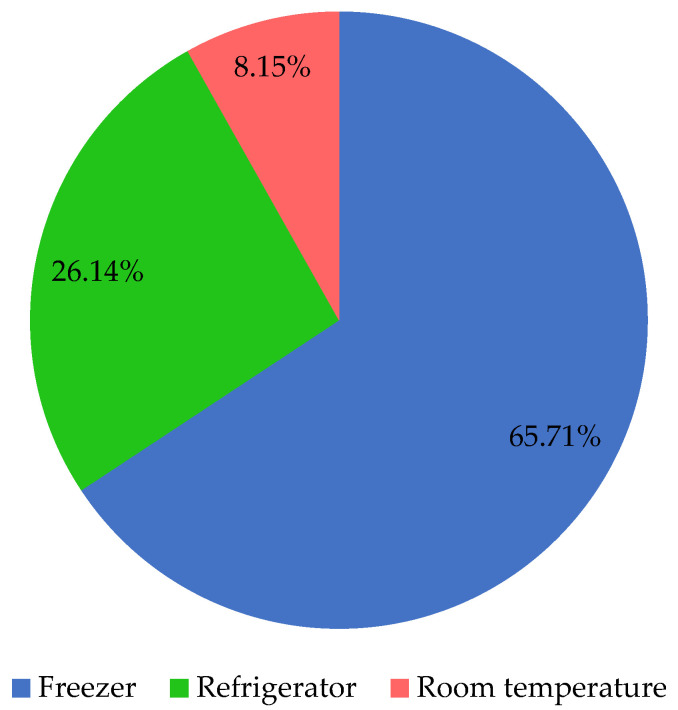
The frequency of different bread storage methods among the surveyed households.

As Figure 1 shows, almost two thirds of the study population reported that they store bread in freezers, while about one fourth use refrigerators. Storing bread at room temperature was found to be the least common way of storing bread in the surveyed households.

**Figure 2:**
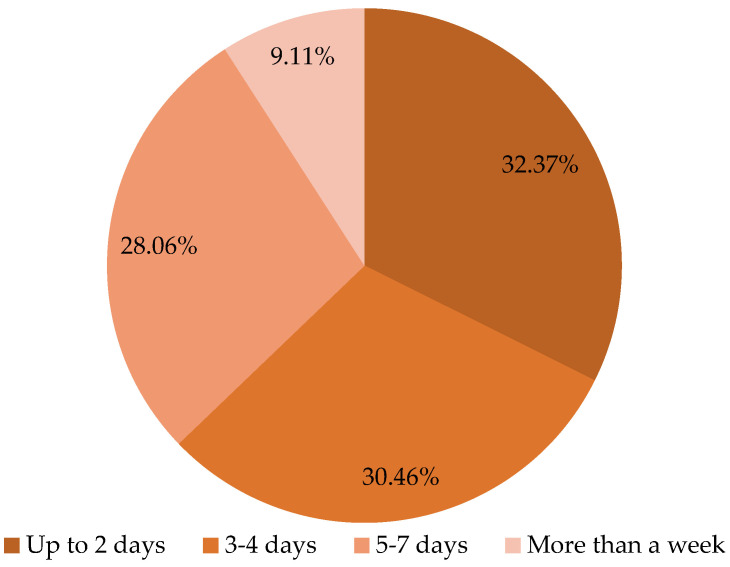
The presentation of different bread storage duration groups.

Figure 2 indicates that almost a third of the respondents stated that, in their households, bread is normally kept up to two days after purchase. Less than a third of the study population stated their bread storage duration is about 3–4 days, followed by the group who reported storing bread for 5–7 days after purchase. A minority reported that they store bread for more than a week.

Linear regression models revealed that the storage method had a significant effect on the total HBW (α = 0.026) and TB waste (α = 0.000) and that storage duration and its interaction with storage method did not affect total HBW. Neither storage method nor duration caused any significant variation in NTB waste data.

Table 4 compares the mean waste percentages of the bread categories, which are presented based on the storage methods. The pairwise Bonferroni post hoc test revealed that for the total and TB waste there is a significant decrease in wastage when bread is stored in freezers and refrigerators compared to storage at room temperature. The NTB waste mean values did not significantly differ among the storage method groups.

### 3.4. Previous Publications about HBW in Iran

The document review identified seven publications and reports focused on HBW in Iran. Table 5 lists these documents and summarizes their findings and methodologies. These documents were published between 1991 and 2015. The oldest publication was a national research project report by Mirfakhrayi [42] investigating bread waste in households and bakeries in Tehran, Iran, by employing a direct measurement method. Similar research was conducted in 2015 by Irani et al. [43], who also used direct measurement to assess HBW in the provinces of Tehran, Khuzestan, and Golestan. Moreover, the Iranian parliament published two reports on HBW in 2012 and 2015. Only one peer-reviewed article [44] focused on HBW in Iran. The two other publications were presented at national conferences [45,46]. As can be seen, only Irani et al. [43] stated that HBW in Iran ranges between 12 and 16%, while others claimed that HBW in Iran is around 30%. Among all reports, only two were published based on primary data collection.

## 4. Discussion

This study revealed that almost all of the study population consume at least one type of TB, and almost 75% purchase one or more type of NTB. This privileged the study by enabling the participants to easily relate to the HBW questions. The results showed that the mean waste of all bread types in Shiraz was 1.80%, ranging from 1.00–3.58%, depending on the bread type. Based on the documents we found, both the TB and NTB waste figures in our study were substantially lower than the 30% that is widely regarded as the amount of HBW in Iran [42,44,45,46,47,48]. However, the reference or the methodology for some of these reports seem ambiguous or, in a few cases, not even accessible, and actual field studies to quantify HBW in Iran are scarce. Among the authors who claimed that the HBW is around 30%, only Mirfakhrayi et al. [42] carried out a primary data collection using the direct measurement method. However, this study took place 30 years ago and may, therefore, no longer be applicable. Between the two reports by the Iranian parliament in 2012 and 2015, the first one cited Mirfakhrayi et al. [42], and the latter did not mention any reference. From the other three publications, Omidvar et al. [44] cited Mirfakhrayi et al. [42], Mojarad [46] did not provide any supporting evidence, and Rastegary [45] referred to five reports and interviews with officials that were published by news agencies [49,50,51,52,53,54]. Of those five news releases, one was unavailable on the internet [51]. After factual analysis, we concluded that the accuracy of the officials’ statements in the other four news releases is doubtful. Presumably, those officials referred to the 2012 Parliament report, which cited Mirfakhrayi et al.’s 1991 study that is arguably outdated. Shahedi [55] critically questioned the 30–35% HBW values reported by the officials and conjectured that a more precise estimate at the consumption level in Iran should be around 20% HBW.

The most recent and the only other primary data collection that we found was carried out by Irani et al. [43], who found the HBW to be 12–16% using direct measurement. Although our findings are in better agreement with Irani et al. [43], the difference is still inordinate. The following explanations could be argued to justify or elucidate why our HBW results were so much lower than the former findings. We oriented our focus mainly on the two studies by Mirfakhrayi et al. [42] and Irani et al. [43], who carried out primary data collection.

The inconsistency in food waste definition could be one reason for the difference between our findings and the previous reports on HBW in Iran. Neither of the two studies that carried out primary data collection for HBW assessment in Iran [42,43] provided a clear definition for HHFW in their papers. As stated in Section 2, the definition provided by Corrado and Ardente [38] was used in this study, which excludes portions of edible material used for other beneficiary purposes—e.g., cooking, feeding domestic animals, and compost. In other words, if the household used not-eaten food for other purposes, that amount did not count as waste. Therefore, since it is unclear whether Mirfakhrayi et al. [42] and Irani et al. [43] included the not-eaten portions of food used otherwise in their waste calculations, it is difficult to compare their results with ours. The contrast in HHFW definitions in various studies has been a crucial obstacle in comparing data across studies [56]. Nonetheless, the dichotomy between our findings and the previous ones remains considerable.

Another reason for the deviation of our findings from previous ones could be sought in the nature of the method used in this study to assess HBW, which was HHFW self-assessment by means of recall questionnaire. There is ample evidence suggesting that self-assessment methods for estimating HHFW—including recall questionnaires—underestimate the waste amount [10,57,58,59]. The majority of respondents claimed that no bread wastage occurs in their households. More than half of the TB consumers reported that they do not waste TB, while two-thirds of the NTB consumers reported zero waste for that bread category. Our observation bears a close resemblance to Djekic et al.’s [60] study on HHFW in Serbia in which a high number of respondents were zero wasters, ranging from 37.2% of the respondents for bread and bakery products to 78.1% for processed fruits. However, responses to recall questions may contradict the truth, as in self-assessment the participants may understate the food waste amount because they might not recall the precise amount or even the occurrence of wastage [61]. Some participants’ dishonesty in answering the questions or their embarrassment in admitting to food wastage could also cause an underestimation of food waste [62]. Therefore, it seems safe to assume that our waste results were lower than the actual amounts in the studied households, partly due to the employed methodology.

The underestimation ratio (UR) for HBW assessment of the same study population was calculated to be 1.80, 1.24, 1.58, and 1.46 for sangak, baguette, bun, and sandwich bread, respectively [63]. The UR is the ratio of the more accurately estimated waste results to the outcome of the self-assessment questionnaire. The accurate HBW values in Ghaziani et al.’s [63] work were calculated by measuring HBW at a lab after replicating the waste-causing consumption recipes. In order to correct the underestimation bias in our data, the sangak UR can be used for the TB waste values, and the average of the other three URs can be used for the NTB. As a result, TB waste would turn out to be 3.06%, and NTB waste would become 3.58%. These figures would likely reflect a more accurate estimation of HBW in the surveyed households. Nonetheless, the outcome would still be substantially lower compared to previous findings.

Another possible explanation for achieving different outcomes may be the change in bread storage method over time. The analysis of our data showed that storing at room temperature causes more waste as opposed to storing in freezers or refrigerators. Nevertheless, no association was observed between the storage method and NTB waste. However, based on our anecdotal observations, the surveyed households mostly stored TB for longer and consumed NTB fresh. As the question regarding storage method was asked regardless of the consumed bread type, it could be assumed that the respondents were referring to the TB storing method while answering the question. Given that assumption, it is reasonable to conclude that the storage method does not affect NTB waste. However, the storage method effect on total HBW and TB waste remains relevant. Mirfakhrayi et al. [42] stated that, among their study population, 9.6% used freezers, and 42.3% used refrigerators for bread storage, while almost half of the participants reported that they keep bread at room temperature. Contradictorily, a small minority of our interviewees indicated they store bread at room temperature, and more than 90% used either freezers or refrigerators. This change could be associated with the positive trend in Iranians’ access to modern appliances such as freezers and refrigerators over the last few decades [64]. Unfortunately, Irani et al. [43] did not include the storage method in their research; therefore, we could not obtain information to enable the comparison of bread domestic storage methods between Mirfakhrayi et al.’s [42] work and ours.

In addition, the storage duration effect was investigated in the present study. However, the data analysis did not reveal any significant impact in that respect. Despite the possible assumption that due to a higher storage length a higher bread wastage occurs, duration alone is not responsible for bread decay and wastage, and the more determining factor is the storage method. For example, bread can be stored for a long period in a freezer without causing additional waste. Therefore, solely analyzing the effect of bread storage duration on HBW is irrelevant, while further investigations on the interaction effect of storage method and duration are required. Such an interaction effect was not found in our dataset, possibly resulting from the small sample sizes in the data subgroups.

Furthermore, the relatively low HBW in this study could be attributed to the economic recession and the spike in bread prices shortly before and during our survey. Concurrent with our study, Iran was suffering from an unprecedented economic crisis. Iran’s economy faced enormous shocks and turbulences during the 2016 US presidential election, after Donald Trump’s victory in the election, and after his withdrawal from the Joint Comprehensive Plan of Action (JCPOA) [65]. The Trump administration started imposing new sanctions on Iran in July 2017, followed by other sanctions and opposing measures, with the climax being America’s withdrawal from the JCPOA in September 2018 [66]. The aftermath soon evolved into drastic impacts on Iranians’ livelihood. The annual growth in the consumer price index ranged between 6.4% and 18.2% from 2012–2017. This index raised to 29.1% and 47.8% in 2018 and 2019, respectively [67]. The point-to-point inflation rate was almost doubled in October 2018 (18.4%) compared to 9.9% in the previous year [68]. The Iranian currency value fell drastically [69], and the country’s oil export decreased continuously during Trump’s presidency [70]. Consequently, the price of a vast majority of commodities, including food, rose dramatically.

The pace of price growth for food commodities, including bread, came to a tremendous high point in December 2018, coinciding with the start of our survey. According to a report published by the Central Bank of the Islamic Republic of Iran [68], in November 2018, the food consumer price index witnessed a 59.9% increase, with the bread index rising 31.3% compared to the similar month of the previous year. For food and bread indices, these numbers were 14% and 10.9% in November 2017, respectively, [71]. The same report indicates a sharp surge in the slope of the consumer price index changing trend, particularly for the food group index, occurring around the middle of 2017, testifying to the drastic impact of the sanctions and political complications on food prices. During this period, the income of Iranian urban households increased by only 18.6% in 2018 [72] and 24.4% in 2019 compared to the corresponding preceding year [67].

As a result, Iranian consumers’ purchasing power decreased enormously in 2018 and 2019 [73]. Several studies have shown that higher food prices could lead to less food waste [74,75,76,77]. This correlation may well be a consequence of consumers avoiding over-purchasing when food prices rise [74,75]. A lower purchasing power may encourage consumers to adopt a more frugal lifestyle and motivate them to avoid food waste to reduce monetary loss [78,79]. Therefore, the Iranian consumers may have chosen a more conservative approach to food purchasing while trying to utilize their resources with caution, which leads to lower HHFW—including HBW.

The bread quality improvement during the last decade in Iran could also be deemed an influential factor in HBW reduction. A national policy that came into effect in 2010 focused on improving the quality of bread production and consumption [80]. Since 2013, the Iranian Ministry of Health and Medical Education has allocated an expert working group to promote the nutritional quality of bread [81]. The evidence suggests that low food quality could be one of the drivers of wasting food at the consumption stage [82,83,84]. Therefore, there is a logical relationship between the recent bread quality improvement and the limited HBW found in this study.

Culturally speaking, bread is highly revered among Iranians. Many Iranians refer to bread as “God’s blessing,” and they consider it “holy” [26]. Therefore, wasting bread is stigmatized among Iranian consumers, and it would be rational to assume that bread waste in Iranian households is limited. Other studies may be found that indicate a relatively low amount of loss or waste for other commodities. For example, Tostivint et al. [85] estimated a 1.4% loss throughout Pakistan’s dairy supply chain stages, including suppliers, collection points, dairy factories, and distribution and retail. In another study, Silvennoinen et al. [86] (p. 1061) reported that most of the participants in the Finnish households “produced little food waste”—less than 1 kg of food waste within two weeks. Nonetheless, they could not provide a percentage of total purchased food waste, as this related question was not asked. In general, consumers feel responsible for food consumption and show ethical and social concerns about food wastage [87,88]. In particular, the NENA countries that are known to have predominantly Muslim populations are expected to avoid wasting food, as it contradicts the teachings of Islam [16]. Nonetheless, HHFW remains a major issue in that region. This issue cannot be effectively addressed unless an accurate estimation and evaluation of the level, reasons, and hotspots of food wastage are obtained.

Overall, our findings confirm the UNEP’s [4] very low confidence in Iran’s HHFW official statistics. Nonetheless, HBW in Iran may not be the only example of contradictory information. The UNEP recognizes the confidence level of most NENA countries’ HHFW data to be either low or very low [4]. However, the ambiguity about HHFW statistics is not limited to NENA or even developing countries. As explained in Section 1, only 39 countries continuously reported on FLW to the FAO [2], and the UNEP recognized the data provided by only 17 countries to be of desirable quality [4]. Koester and Galaktionova [56] also discuss the example of FWL in the Russian Federation and conclude that the official FLW data available at the Russian Federal State Statistics Service (Rosstat) are widely misrepresented and are based on approximate estimations. Another example would be the inconsistent FLW definitions between the United States Environmental Protection Agency (USEPA) and the United States Department of Agriculture (USDA), which may lead to a misleading presentation of their national FLW data [89].

Based on the preceding arguments, there is an urgent need for policymakers—especially in developing countries where the main data gap exists [10]—to re-evaluate their statistics’ accuracy and keep their data up-to-date. Therefore, allocating proportional academic budgets to conduct further research on HHFW in NENA seems to be a rational investment that would increase agri-food systems’ efficiency while reducing environmental and economic burdens and improving food security and social well-being.

Although obtaining data at a national level would be ideal, the value of sub-national data cannot be underestimated. Our study focused on one food commodity in one city, yet its contribution remains significant. As described in Section 3, Mirfakhrayi et al.’s [42] work that the officials widely cite was also carried out in one city in Iran. HHFW data availability has been persistently expanding, mainly owning to sub-national studies [4]. Policymakers can obtain more pieces of the multifaceted HHFW puzzle by increasing the number of studies at the city and municipality level, the inclusion of which would provide a more accurate evaluation of the status quo.

Meanwhile, it is crucial to implement the latest developed definitions and methods to allow for comparison across studies and geographical settings [90]. For this reason, the UNEP developed the Food Waste Index (FWI) to establish “a consistent approach to monitor SDG Target 12.3” [91] (p. xiii). Although this index still might have some shortcomings—e.g., neglecting the economic value of different commodities’ waste [56]—they facilitate the comparability of data across studies and improve the reliability of future data.

Obtaining reliable first-hand data, among other things, would remain a principal challenge ahead of governments and international organizations such as the UNEP for drawing up estimates on food waste. Enhancing statistical knowledge should be an underlying priority for the international community to assess the progress in achieving the SDGs [2]. In the international workshop for capacity building for FLW reduction in the Near East in 2017, enhancing data collection and analytical methodologies was emphasized as the first component for achieving SDG 12.3 [92]. However, as Xue et al. [10] shrewdly stated, gathering data on the quantity of food waste is the first step, and developing effective policies and plans to monitor and reduce food waste must be prioritized.

## 5. Conclusions

Assessment of HBW in Shiraz revealed an extensive deviation from the official reports in our findings. Due to limited research capacity, the present study focused only on one food commodity in one city. Therefore, the waste results may not be generalized with regard to other food commodities and other locations. Moreover, as discussed in Section 4, the quantification method employed in this study bears a substantial level of underestimation, which may undermine the reliability of our data. However, despite the possible shortcomings and limitations, this study is one of the rare investigations on HBW in Iran based on primary data collection, resulting in detailed datasets. According to the present study, there is a substantial likelihood of bias in the HHFW data that are provided by local governments. As the UNEP generates a major share of its estimates based on the extrapolation of these data, it is crucial to examine whether countries’ claims on HHFW are backed up by ample evidence. Obviously, additional studies on HHFW, especially in developing countries—where the main data gap exists—are necessary in order to gather more data that are compatible with SDG 12.3. The governments should invest in more studies to collect first-hand, up-to-date data to be able to develop effective food waste reduction policies and courses of action. Moreover, we recommend that the UNEP should undertake further empirical research to cross-check and examine the reliability of the data provided by officials and governments. This study reaffirms the necessity of treating the HHFW data that are already available with great caution.

## Figures and Tables

**Table 1:** The description of the studied bread types based on their categories and characteristics.

Bread Category	Bread Type	Description		
		Geometric Shape	Bread Kind	Texture
Traditional	Lavash	Rectangle, pseudo-ellipse	Non-sweet, flat	Soft, crispy
	Sangak	Pseudo-triangle	Non-sweet, semi-raised	Crispy
	Taftoon	Circle	Non-sweet, flat	Crispy
	Traditional babari	Pseudo-ellipse	Non-sweet, semi-raised	Crispy, soft
Non-traditional	Baguette	Pseudo-ellipse	Non-sweet, raised	Crispy
	Hamburger (bun)	Circle	Non-sweet, raised	Doughy
	Sandwich	Pseudo-ellipse	Non-sweet, raised	Soft, doughy
	Broetchen	Pseudo-ellipse	Non-sweet, raised	Doughy
	Toast	Rectangle, square	Non-sweet, semi-raised	Soft
	Non-traditional barbari	Pseudo-ellipse	Non-sweet, raised	Soft

Source: Adopted from Karizaki’s [26] and the Iranian National Standardization Organization [36,37].

**Table 2:** Demographic and socioeconomic summary of the studied households.

Variables		N	% of Total N	Mean	(SD)
Gender ^1^	Female	399	95.20	48.23	(13.41)
	Male	20	4.80	48.20	(20.52)
Head of the household	Female	39	9.31	54.74	(15.03)
	Male	380	90.69	47.56	(13.51)
Education ^2^	Illiterate and primary	94	22.40	53.64	(13.86)
	High school and diploma	229	54.70	46.75	(12.68)
	University degree	96	22.90	46.46	(15.03)
Occupation ^2^	Unemployed	49	11.70	55.24	(14.38)
	Skilled worker	105	25.10	43.03	(12.15)
	Employee	116	27.70	42.29	(12.87)
	Professional	23	5.50	42.30	(11.49)
	Retired	126	30.10	56.37	(10.51)
Total		419	100.00	48.23	(13.8)

N = Number of observations; SD = Standard deviation; ^1^ Variable belonging to the respondent; ^2^ Variables belonging to the head of household.

**Table 3:** The presentation of bread waste percentages based on bread types and categories.

Bread Types and Categories	N	Waste Mean Value (%)	SD
Lavash	335	1.96	5.31
Sangak	399	1.89	4.74
Taftoon	47	1.37	2.83
Traditional babari	197	1.57	5.05
Traditional bread	416	1.70	3.70
Baguette	149	3.58	6.72
Hamburger	136	2.54	5.25
Sandwich	229	2.97	7.04
Broetchen	86	3.43	8.46
Toast	132	3.46	10.31
Non-traditional barbari	54	1.00	3.50
Non-traditional bread	304	2.50	5.26
Total	416	1.80	3.36

N = Number of observations; SD = Standard deviation.

**Table 4:** Mean waste percentages of different bread categories based on storage method.

Storage Method	Freezer	Refrigerator	Room Temperature
Total waste	1.62 ^a^	1.81 ^a^	3.43 ^b^
N	273	109	32
Traditional bread waste	1.31 ^a^	1.91 ^a^	4.36 ^b^
N	273	109	32
Non-traditional bread waste	2.43	2.51	2.52
N	197	78	28

N = Number of observations. Note: The values with different superscript letters in a row are significantly different (*p* < 0.05). The group sizes are unequal. The harmonic mean of the group sizes is used.

**Table 5:** The list and description of publications about household bread waste in Iran.

Publication Year	Author(s)	Stated BW	Method/Reference	Publication Type
1991	Mirfakhrayi et al.	33.5%	Direct measurement at HH level	Research project report
1994	Mojarad	30%	Not given	Conference paper
2005	Irani et al.	12–16%	Direct measurement at HH level	Research project report
2007	Omidvar et al.	30%	Mirfakhrayi et al. [42]	Peer-reviewed article
2012	Baradaran Nasiri et al.	33.1%	Mirfakhrayi et al. [42]	Parliament report
2015	Talebi	20–40%	Not given	Parliament report
2015	Rastegary	30%	News agencies	Conference paper

BW = Bread waste; HH = Household.

## Data Availability

The data presented in this study are available on request from the corresponding author. The data are not publicly available due to privacy restrictions.
